# Degradation of Polyvinyl Alcohol in US Wastewater Treatment Plants and Subsequent Nationwide Emission Estimate

**DOI:** 10.3390/ijerph18116027

**Published:** 2021-06-03

**Authors:** Charles Rolsky, Varun Kelkar

**Affiliations:** 1 Biodesign Center for Environmental Health Engineering, The Biodesign Institute, Arizona State University, 1001 S. McAllister Avenue, Tempe, AZ 85287, USA; vpkelkar@asu.edu; 2 Plastic Oceans International, Malibu, CA 90265, USA; 3 School of Sustainable Engineering and the Built Environment, Arizona State University, 660 S. College Avenue, Tempe, AZ 85281, USA

**Keywords:** biodegradation, dish detergent, laundry, mass loads, microbes, polyvinyl alcohol, wastewater

## Abstract

Polyvinyl alcohol (PVA) is a water-soluble plastic commercially used in laundry and dish detergent pods (LDPs) for which a complete understanding of its fate in the environment and subsequent consequences is lacking. The objective of this study was to estimate the US nationwide emissions of PVA resulting from domestic use of LDPs, corroborated by a nationwide, online consumer survey and a literature review of its fate within conventional wastewater treatment plants (WWTPs). Peer-reviewed publications focusing on the degradation of PVA in critical processes of WWTPs were shortlisted as a part of the literature review, and subsequent degradation data was extracted and applied to a model with a set of assumptions. Survey and model results estimated that approximately 17,200 ± 5000 metric ton units per year (mtu/yr) of PVA are used from LDPs in the US, with 10,500 ± 3000 mtu/yr reaching WWTPs. Literature review data, when incorporated into our model, resulted in ~61% of PVA ending up in the environment via the sludge route and ~15.7% via the aqueous phase. PVA presence in the environment, regardless of its matrix, is a threat to the ecosystem due to the potential mobilization of heavy metals and other hydrophilic contaminants.

## 1. Introduction

**Figure 1:**
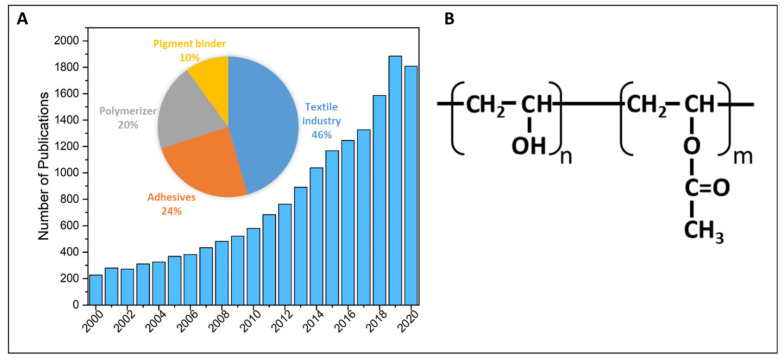
(**A**) Increasing number of publications per year focusing on polyvinyl alcohol (PVA) as well as a pie chart depicting percentage distribution of PVA applications [3] and (**B**) the chemical structure for partially hydrolyzed polyvinyl alcohol-acetate.

Plastic pollution has been steadily increasing since the 1950s [1]. Due to an upsurge in public awareness regarding plastic usage and pollution, more “sustainable” alternatives have increased in popularity, and are thus being utilized in higher quantities by the general public [2]. These new materials are often marketed as “biodegradable”, as they are considered to be susceptible to microbial degradation under specific conditions, but this specificity often makes it difficult to understand their ultimate fate in the environment. Polyvinyl alcohol and its corresponding blends (PVOH, PVAI, Polyviol, Alcotex, Covol, Gelvatol, Lemol, Mowiol, Mowiflex, and Rhodoviol) are examples of polymers that have become more popular both in usage and within scientific research (see Figure 1A) due to their water-solubility. Typically, PVA is used as a protective film for laundry and dish detergents; as a sizing and finishing agent in the textile industry [3]; and as a thickening or coating agent for paints, glues, meat packaging, and pharmaceuticals in paper and food industries (see Figure 1A) [3].

Up to 650,000 tons of PVA is produced yearly across the globe [4] and this has been expected to increase 4.09% annually from 2018 to 2023 [5]. In 2018, due to its general increase in usage, PVA was considered to be one of the most ubiquitous pollutants in wastewater [4,6,7]. A thorough understanding of its path to and breakdown within the environment is presently lacking. Although water-soluble, its constituents, such as ethylene (a petroleum-based product), can remain intact within the solvent. Studies have shown ethylene to have negative effects on surrounding organisms, such as plants, which naturally produce and utilize ethylene [8]. Similar to table salt and sugar, PVA dissolves in water, and if the water volume is low, a viscous solution will be formed. The high water volume in WWTPs means the texture of the water should remain unchanged. When PVA is discharged into water bodies, it has the ability to foam due to its surface properties [9]. This can inhibit oxygen transfer, causing irreparable harm to aquatic life [10]. Additionally, because of its hydrophilicity, PVA has the potential to adsorb dangerous chemicals or contaminants [11], such as antibiotics [12] or heavy metals [13,14,15], at high concentrations. These can then concentrate up food chains [16], posing a threat to the environment, similar to behavior of traditional polluted plastics. WWTPs are known to contain a variety of dangerous contaminants, creating a higher-risk situation for PVA particles passing through [17].

The PVA used in LDPs is composed of PVA polymeric chains with a fraction of polymeric acetate groups (see Figure 1B). This is referred to as partially hydrolyzed PVA, and the percentage of hydrolysis and molecular weights vary with its application [18,19]. The PVA used in LDP blends is typically 88% hydrolyzed [18], while its molecular weight can vary within several ranges, including but not limited to 1000–1,000,000, 10,000–300,000, and 20,000–150,000 Da [19]. Upon contact with water, the presence of polymeric acetate groups enables the water molecules to penetrate the bonds of the outer coating and break it into smaller chains. Once flushed down the drain, partially hydrolyzed PVA chains (See Figure 1B) enter wastewater channels, eventually interacting with WWTPs. The fate of PVA in wastewater treatment systems has been partially explored, with some studies highlighting specific aspects of the wastewater treatment process, but very few studies aimed to establish a complete degradation estimate in conventional WWTP processes from beginning to end.

Available studies suggest that the degradation of PVA occurs under a specific set of circumstances, which may not be ubiquitous within WWTPs or the natural environment. Ultimately, PVA degradation is reported to be a slow process, which greatly depends upon the surrounding conditions mentioned in Table S2 [20]. Bacteria utilize enzymes to degrade PVA to its constituent form by attacking specific bonds within the polymeric chain [7,21]. Bacteria oxidizes the tertiary carbon atoms, leading to the endo-cleavage of PVA molecules, one of the main degradation routes, which leads to the creation of byproducts such as hydrolyzable hydroxy ketone and 1,3-diketone [20,22]. Other microorganisms mainly utilize PVA as a carbon source; such is the case with the bacteria *Pseudomonas* [20], which generate hydrogen peroxide and other byproducts, including a lower molecular weight PVA [23,24]. Many of these processes can take place simultaneously to begin the degradation of the polymer [20]. While several bacterial species have been documented degrading PVA, these are infrequently found within conventional WWTPs or the environment [20], as are the other optimal circumstances necessary for PVA to completely degrade (Table S1). WWTPs are predominantly designed to remove suspended solids, harmful bacteria, and pollutants of emerging concern [25], but the removal of PVA is still in question due to a lack of comprehensive research. Independent studies have been performed demonstrating removal of PVA using different bacteria, enzymes, and chemical processes [20], but a complete fate assessment in a conventional WWTP or the environment is currently lacking.

In this study, we present a detailed qualitative, quantitative, and spatial analysis on annual mass emissions of PVA in the US, consisting of three defined segments: (i) an online survey involving 500+ respondents investigating their laundry and dish detergent purchasing habits (ii) a United States Geological Survey (USGS) report on water usage, sources, and population; (iii) and a detailed literature review on the degradation of PVA in wastewater, utilizing the data to conduct a subsequent mass balance analysis. 

## 2. Materials and Methods

### 2.1. Online Survey 

An online survey was conducted with 527 respondents, 80% female and 20% male, ages 24–55 years. The survey was directed at the primary decision-maker responsible for purchasing cleaning products in the household. In total, 60% of the responses were from the top 20 designated market areas (DMAs). Survey questions mainly investigated the frequency of buying laundry and dish detergent and the type of detergent bought (single dose pods or detergent bottle) (see Supplemental Information). 

### 2.2. Water Use and Wastewater Generation in the US

**Figure 2:**
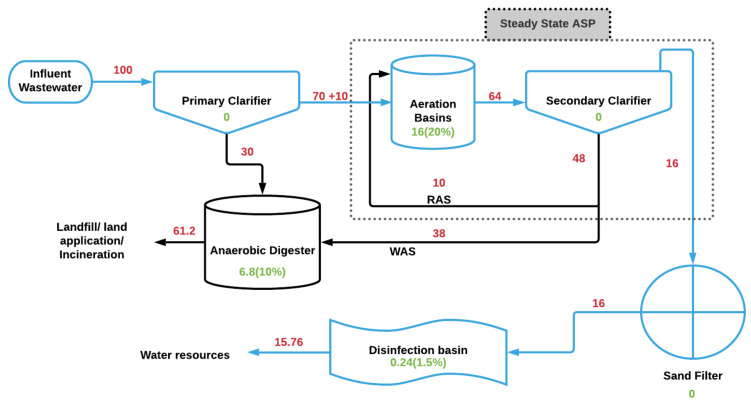
Mass balance of PVA in a conventional activated sludge treatment plant, considering clarifier efficiencies and biodegradation efficiencies. Numbers in red indicate the percentage of PVA in respective treatment streams, and numbers in green represent the amount (% absolute) of degraded PVA in respective sections. RAS and WAS represent return activated sludge and waste activated sludge, respectively. Numbers in parentheses represent the degradation efficiencies of respective sections.

State wise data on water use and application for the United States was published in the USGS report titled “Estimated Use of Water in the United States in 2015” [26]. Treatment plants in the US receive wastewater from public facilities, domestic sources, and industrial effluents. Hence, the total water used that ultimately resulted in the generation of wastewater was the sum of public, domestic, and industrial supply (Mgal/d) as shown in Equation (1) which assisted in populating Figure 2.

(1) $$W_{Use,2015}=W_{P}+W_{D}+W_{I}$$

In this equation, *W_P_*, *W_D_*, and *W_I_* stand for public, domestic, and industrial water supplies, respectively.

Not all water used is received by WWTPs as wastewater; there are an estimated 20%–25% in losses [27]. which include but are not limited to leakages of sewers and sanitary sewage outflows. 

The total wastewater generated was calculated using Equation (2), assuming a 20% loss in volume. In this case, *WW_G,_*_2015_ stands for the wastewater generated for each state.

(2) $$WW_{G,2015}=0.8*\left(W_{P}+W_{D}+W_{I}\right)$$

Roughly 16,000 US WWTPs treat 34 billion gallons (*BGD*) of wastewater daily [28]. This represents an average of approximately 2.12 billion gallons treated daily per facility. Wastewater treated for each state can be calculated using Equation (3), where *N_F_* and *WW_T_*_,2015_ represent the number of wastewater treatment facilities in each state and amount of wastewater treated, respectively, assuming a complete use of operational treatment capacity.

(3) $$WW_{T,2015}=N_{F}*2.12\;BGD$$

The untreated wastewater was calculated as the difference between generated and treated wastewater, as shown in Equation (4). Untreated wastewater could be a result of several possibilities, including but not limited to the lack of connectivity between households and WWTPs, improper disposal of wastewater generated, and/or generated wastewater exceeding the operational capacity of a WWTP (current assumption).

(4) $$WW_{UT,2015}=WW_{G,2015}-WW_{T,2015}$$

Subsequently, the percentage of wastewater untreated and treated were calculated as shown in Equations (5) and (6) respectively.

(5) $$WW_{UT,\%}=\frac{WW_{UT,2015}}{WW_{G,2015}}\;*\;100$$

(6) $$WW_{T,\%}=\frac{WW_{T,2015}}{WW_{G,2015}}\;*\;100$$

### 2.3. LDP Usage and Treatment

Laundry pods from three brands and dish pods from two brands were drained, air-dried overnight, and weighed on an Ohaus Adventurer weighing scale (AR1530, China). Laundry pods from different brands (*n* = 9) and dish pods from varying brands (*n* = 6) were weighed in triplicate. The average weights for laundry (*M_L_*_,*Avg*_) and dish (*M_D_*_,*Avg*_) pods were 1.0 ± 0.6 g and 0.5 ± 0.2 g, respectively. 

Data from the online survey revealed consumption of ~15 billion laundry pods (*N_L_*_,*Avg*_) and ~12 billion dish pods (*N_D_*_,*Avg*_) per year by 126 million households in the US. Using 2015 state-wise population numbers from the USGS report [29] (*P*_2015_), the number of per capita pods consumed in the US (*N_PC,Avg_*) was calculated using Equation (7).

(7) $$N_{PC,Avg}=\frac{N_{L,Avg}+N_{D,Avg}}{P_{2015}}$$

The outer dissolvable coating is composed of PVA and other additives in varying proportions. Based on patents and reports, the PVA ratio (by weight) lies between 65% and 99% of the total outer coating weight [30]. An average mass of PVA in laundry and dish pods was calculated using Equation (8), where *f_L_* and *f_D_* are the fractions of PVA in laundry and dish pods, respectively, in grams.

(8) $$M_{PVA,Avg}=M_{L,Avg}*f_{L}+M_{D,Avg}*f_{D}$$

The number of pods (laundry and dish) used by each state was calculated as per Equation (9).

(9) $$N_{Pod}=N_{PC,Avg}*P_{2015}$$

The mass of discarded PVA from laundry pods (*M_L,G_*) and dish pods (*M_D,G_*), in each state was expressed as per Equation (10).

(10) $$M_{PVA,G}=N_{Pod}*M_{PVA,Avg}$$

Untreated and treated masses of PVA were calculated as per Equations (11) and (12), respectively.

(11) $$M_{UT,PVA}=M_{PVA,G}*\;WW_{UT,\;\;\%}$$

(12) $$M_{T,PVA}=M_{PVA,G}*\;WW_{T,\;\;\%}$$

Applying total degradation percentages (solid + aqueous phase) from the modeled scenario to the PVA treated (*M_T,PVA_*) would result in total PVA emissions from WWTPs.

### 2.4. GIS and Mapping 

**Figure 3:**
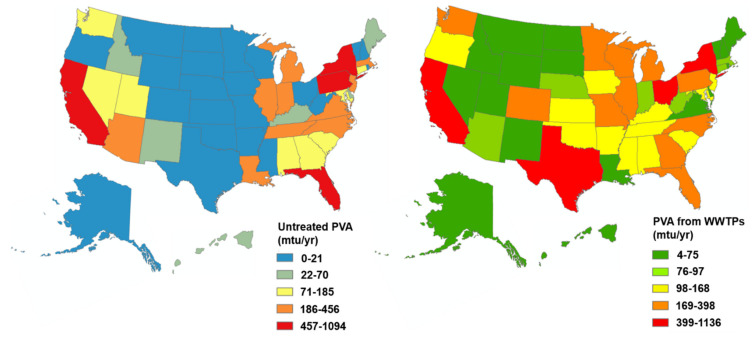
PVA emissions across the U.S. in mtu/yr. The left panel is the spatial distribution of untreated PVA via wastewater that does not reach the treatment plants. The right panel represents the PVA from WWTP effluent streams, including aqueous and sludge disposal routes.

Figure 3 was created in Arc GIS pro 2.0.0. Data specific to the US states and environmental emissions of PVA (metric tons/yr) were collected from outside sources. These were then imported into the GIS software program ArcMap. 

### 2.5. Literature Review

A comprehensive literature review was performed on the presence and degradation of PVA in US wastewater according to PRISMA guidelines. A detailed breakdown of the publication selection is presented in SI Figure S1. Databases accessed included Google Scholar and Scopus, utilizing the search terms polyvinyl alcohol*; PVA*; Polyvinyl alcohol with results further specified using the constraining term (AND): Pollution*; Degradation*; Biodegradation*; Wastewater*; WWTP*; Sludge*; Sewage*; Effluent*; Influent*; Activated sludge*; US. Papers published between 1950 and 2020 were sought out. These studies were specifically sought out as they aimed to assess the breakdown of PVA within a conventional WWTP by microbial attack or other forces, such as UV radiation or chlorine oxidation. From each relevant paper, the degradation mechanism was extracted as well as the percent degradation, WWTP section (if applicable), the microorganism species, and whether or not the species was adapted to the wastewater itself. As many of the papers included several independent studies relating to PVA degradation in wastewater, a range of percentages were then included to encapsulate all reported values within a single manuscript. Papers with incomplete text or a lack of relevance to wastewater were excluded (see Figure S1).

## 3. Removal of PVA in WWTPs

The majority of the PVA, generally in the form of greywater from domestic, public, and industrial sources, reaches WWTPs, where it encounters primary treatment, secondary treatment, sludge treatment, and disinfection before leaving the WWTP. 

### 3.1. Primary Treatment

Primary treatment consists of initial screening, grit removal, and a primary clarifier, with the objective of removing coarse solids and other large items [31]. PVA has not been studied in the context of its partitioning and removal in primary treatment within a conventional WWTP. Degrading PVA is a challenge and can contribute to the total chemical oxygen demand (COD) [32] from the incoming wastewater. The most influential mechanism of removal in a primary clarifier is the sorption to the suspended solid particles [33]. However, PVA is a hydrophilic polymer and has a greater affinity toward water, tending to stay in the liquid phase as opposed to solid [34]. It is possible that PVA could form gelatinous consistencies with fats and lipids from influent wastewater, which may increase its partition toward solid matter and its removal from the aqueous phase. An empirical study analyzing hydrophilic pharmaceuticals and endocrine-disrupting chemicals in wastewater revealed the low sorption capacity of hydrophilic contaminants to solids, requiring relatively longer retention times (5–11 h) for their efficient removal from the aqueous phase [35]. Current literature evidence is not sufficient to establish a definitive path PVA may take in a primary treatment system, but the COD removal efficiency of the primary clarifiers likely eliminates PVA from the aqueous phase [36]. 

### 3.2. Secondary Treatment

Secondary treatment is designed to further remove organics and suspended solids [31]. Two main components of secondary treatment are activated sludge processing and the use of the secondary clarifier. 

#### 3.2.1. Activated Sludge Process (ASP)

The activated sludge process involves the recycling of the bacteria/microbes present in the sludge, which are sent back into the aeration chambers. These microbes are responsible for the degradation of organic waste and chemicals from the influent wastewater. The bulk of PVA degradation is anticipated to occur at this stage. There are several key factors influencing the biodegradation of PVA in an ASP. One important element is the food-to-microorganisms ratio (F:M), defined as the load of substrate applied daily per unit of biomass [37]. A general F:M ratio lies anywhere between 0.25 and 0.45 [38]. However, an F:M ratio better suited for PVA degradation lies between 0.1 and 0.15 [39]; thus, a higher number of microorganisms is required to fully degrade PVA, compared with conventional domestic sludge. Typical microorganisms may not be able to adequately break down PVA, as the presence of PVA-adapted microbes is necessary for thorough biodegradation of PVA in the ASP [22,39,40]. PVA adaption often requires a lag time spanning over several weeks [22,39], and the ASP can only be PVA-adapted in WWTPs receiving a heavy influx from textile industries, allowing sufficient time for the microbes present at the facility to adapt to the incoming COD. After the lag phase is complete, biodegradation thereafter occurs exponentially [39]. 

The number of PVA-degrading microbes is limited, and their presence is specific to certain environments and environmental conditions. Schonberger et al. discovered that WWTPs consistent with PVA adaption within the ASP achieved 80% degradation in a 7-day period. Alternatively, the unadapted ASP was consistent with only 18% biodegradation within the same time frame [39]. Hoffman et al. observed similar findings when four different blends of PVA were tested in adapted and unadapted sludge. In unadapted sludge, a degradation of 88 ± 9% of incoming PVA was achieved in 187 ± 25 h for four blends, and ~20% was degraded in 25 h [22]. For adapted sludge, 90 ± 5% was degraded in just 29 ± 2 h [22]. The above findings utilized 88% hydrolyzed PVA, the same composition as that used in detergent pod coatings [30,41]. 

The average hydraulic retention time (HRT) in the ASP is approximately 18–24 h, and the sludge retention time (SRT) is 12–15 days [42]. Due to the hydrophilic nature of PVA, the majority of PVA is expected to be in the water phase, in which the HRT would subsequently play a larger role in its degradation.

#### 3.2.2. Secondary Clarifier

The decomposed sludge mixture then enters the secondary clarifier, where the solid waste is given time to settle, allowing the liquid to enter the tertiary treatment stage. In the secondary clarifier, three removal mechanisms may occur, which include volatilization, biodegradation, and adsorption to solids [33]. The current literature does not present evidence of volatilization, biodegradation, or adsorption of PVA in secondary clarifiers. However, since the majority of secondary sludge contains fluid, and the density of PVA is ~1.2 g/cm^3^, a fraction of PVA may settle and exit via secondary sludge. 

### 3.3. Tertiary Treatment 

Tertiary treatment typically consists of a disinfection chamber and a filtration unit. Older WWTPs use chlorination as an effective disinfectant, along with trickling or gravity filters, due to their moderate efficiency and low cost [43]. These technologies are evolving, as most modern WWTPs rely on advanced oxidation processes (AOPs) and membrane filtration, according to the United States Environmental Protection Agency (USEPA) [44].

### 3.4. Disinfection

In 2017, Ye et al. studied the comparative effects of using UV, chlorine, and a UV-chlorine combination in PVA degradation/oxidation [45]. Their experiments revealed that 20 min of UV irradiation (Is = 2.6 mW/cm^2^) had a minimal effect on the initial concentration of PVA (50 mg/L). Chlorine had a similar result, with just 1.5% of original PVA degraded in 20 min. Chlorine conditions of 20 mg Cl_2_/L and a pH of 7 are synchronous with WWTP disinfection conditions in the US [46]. However, a combination of UV-chlorine treatment resulted in 92% degradation within 10 min and 100% in 20 min. Advanced oxidation technologies have been documented to eliminate PVA completely from wastewater in a matter of minutes [15]. These processes can be expensive and thus not cost-effective for the municipality. 

In 2004, the US EPA reported that chlorination is the most utilized method for wastewater disinfection (EPA primer, 2014). Given the degradation conditions under chlorine alone, only a minimal amount of PVA coming from domestic wastewaters can be expected to be degraded.

### 3.5. Filtration

Sand filtration is one of the most widely used filtration techniques due to its low cost of operation and maintenance [47]. Research on the behavior of PVA in a sand or trickling filter system is lacking. However, in cases of missing data, its fate can be predicted based on the behavior of other commonly used hydrophilic materials, such as pharmaceuticals with an octanol-water partitioning coefficient (log Kow) below 2 (see Table 1). A low log Kow indicates the compound’s affinity toward water and categorizes the compound as hydrophilic in nature. The table below demonstrates the very low removal efficiencies of widely known hydrophilic pharmaceuticals in a sand filtration system. It should be noted that sand filtration had minimal to no effect on the concentrations of the compounds studied, with a mean and standard deviation of −0.08% ± 20%. It is likely that sand filtration would not have a significant effect on PVA concentrations. 

### 3.6. Anaerobic Digestors

Wastewater activated sludge coming from secondary clarifier effluent is treated in an anaerobic digester to reduce the overall volume, destroy pathogens, and control odors [50]. Using anaerobic microbes, the organic matter is further broken down, releasing methane and water as byproducts. This treated sludge, termed “biosolids”, is then safe to be used for agricultural purposes, as an example. In the US, anaerobic digesters are typically catered towards mesophilic (30–37 °C) and thermophilic (50–60 °C) bacteria with retention times of 12–25 and 10–12 days, respectively [50]. 

Matsumura et al. explored PVA degradability using a lab-scale anaerobic digester with activated sludge obtained from a local WWTP in Japan [51]. Two drastically different molecular weights (14,000 and 2000 Da) were selected for the study. In the first 25 days, both PVA blends showed similar biodegradation results of 12.5%. As time progressed, PVA-14,000 degraded at a much higher rate compared with PVA-2000. After 150 days, PVA-14,000 reached 50% degradation, while PVA-2000 was only 37% degraded [51]. Such low biodegradation rates could be attributed to the usage of PVA-unadapted sludge. 

Different PVA-starch blends were studied by Russo et al. under anaerobic conditions. They studied starch to PVA ratios of 90:10, 75:25, 50:50, and 0:100. Anaerobic conditions of 38 ± 5 °C with microbes sourced from activated sludge processes were maintained under nitrogen for up to 100 h. Because PVA used in detergent pods does not contain any starch additives [30], the results of the starch to PVA blend of 0:100 most closely mimicked detergent-derived PVA behavior. The solubilization study yielded lower results for the 0:100 blend, with only 10% of the PVA able to solubilize under anaerobic conditions in 100 h. Its 90:10 counterpart was solubilized up to 60% in the same time allotment [52]. Anaerobic digestion efficacy is often assessed based on the amount of methane and carbon dioxide produced as a byproduct. Upon isolating methane and CO_2_ production analysis, the 0:100 blend produced less than 5 mL/g COD, whereas the 90:10 blend produced ~40 mL/g COD [52]. These results highlight the inefficiency of traditional anaerobic digestion methods for the biodegradation of PVA. 

Another study analyzing the breakdown of PVA-glycerol-starch blends in anaerobic digestors was conducted by Pšeja et al. in 2006 [53]. After 30 days of incubating PVA blends at 35 ± 2 °C with anaerobic bacterial cultures, the percentage of biodegradation was assessed based on the balance of carbon, biogas, as well as the liquid phase [53]. The bacterial inoculum was sourced from unadapted municipal sludge of a local WWTP. A total of 13 blends were studied, including 85%/15% PVA-glycerol, 70%/15% PVA-starch, and a 75%/15%/10% PVA/starch/glycerol blend. The biodegradation percentages varied from 4.1% to 19.8%. The 85%/15% PVA-glycerol blend degraded the least, at 4.1% in 30 days, whereas the 75%/15%/10% PVA/starch/glycerol blend degraded the most, at 19.8% [53]. According to the authors, high degradation rates (high carbon differential) can be attributed to the biodegradation of starch, not the PVA itself. Since detergent pods do not contain starch additives, high biodegradation rates may not be observed during anaerobic digestion. 

## 4. Estimated Mass Balance 

The mass balance presented here is a combination of degradation percentages adopted from Section 3.1, Section 3.2, Section 3.3, Section 3.4, Section 3.5 and Section 3.6, as well as a WWTP scenario assumed by Garrido et al. in 2013 [54]. Additionally, WWTP assumptions similar to those of Garrido et al. regarding primary and secondary clarifier COD removal efficiencies were also adopted. A variety of scenarios can be suggested based on a high number of variations in treatments across the US. However, many US WWTPs are older in age and rely on conventional treatment technologies. A conventional activated sludge facility (completely mixed) with primary treatment (screening, grit removal, and primary clarifier), secondary treatment (aeration basins, secondary clarifier, and activated sludge process), tertiary treatment (disinfection and sand filtration), and anaerobic digester was assumed for this study. The COD removal efficiency of the primary clarifier was fixed at 30% and 75% for the secondary clarifier [54]. PVA was considered to be a part of the total COD in this model, and the efficiencies signifying the percentage of the partitioning of COD within the clarifiers were directly applied to PVA. Table 2 indicates the treatment section and the corresponding degradation percentages for the SRT, HRT, and other process conditions based on the literature review within this study. PVA in the influent wastewater entering primary treatment is considered to be 100%. As PVA passes the primary clarifier, 30% is expected to partition into the solid phase and eventually reach the anaerobic digester. The remaining PVA (70%) in the aqueous phase then enters the activated sludge system (see Table 2). 

The microbial activity in the aeration basins degrades 20% of the PVA in the presence of unadapted sludge during 18–24 h of HRT. Residual PVA (64%) enters the secondary clarifier, where 75% (48% of total) is partitioned into the sludge phase, and 25% (16% of total) is carried over via the liquid phase to the sand filters. The 48% in the sludge phase is further divided into 21% (10% of total) as return activated sludge (RAS) and 79% (38% of total) as waste activated sludge (WAS). Therefore, 10% of the total PVA is assumed to be in the form of return activated sludge (RAS). RAS values are adopted from the EPA report, in which RAS flow varied between 15% and 127% of the secondary influent flow, with the number eventually settling in the lower 20s [55]. RAS and primary effluent amount to 80% of the total PVA entering the aeration basin.

Solid phase effluent from the primary clarifier (30%) and WAS (38%) are treated in an anaerobic digester, where 10% is degraded (6.8% of the total) and 61.2% of the total is left untreated and ready to be landfilled, land applied, or incinerated. 

The aqueous phase, containing 16% of the total PVA reaching the sand filtration stage, enters unaltered into the disinfection basin, where 1.5% is degraded (0.24% of the total) and 15.76% remains intact within the aqueous phase. A detailed mass balance can be found in Figure 2. References for degradation percentages and respective retention times are listed in Table 2.

## 5. Nationwide PVA Emissions via WWTPs (Effluent + Biosolids)

Referencing the above model, it is estimated that ~61.2% of PVA is emitted via sludge, and ~15.7% is emitted through effluent, with ~77% of the PVA still intact after passing through conventional wastewater treatment (see Figure 2). Once PVA has passed through conventional water treatment, either untreated or within sludge, its journey and fate become important to understand. By accessing data relevant to sewage treatment in US states, we were able to project the amount of intact PVA emitted by each US state. Our research indicates that the Midwest of the US has the lowest amount of untreated PVA, possibly due to lower populations and fewer population-dense areas (see Figure 3, left panel). The Southern and some Western areas of the US have lower to moderate volumes of PVA emissions. However, states such as California, Florida, New York, and Pennsylvania have the highest loadings via untreated wastewater. This may be due to the presence of more metropolitan cities and overworked public facilities as populations grow, causing urban expansion [56].

PVA emissions via WWTP effluent demonstrated slightly different data, with certain regions remaining the same (see Figure 3, right panel). Similar to untreated PVA emissions, much of the Midwest had lower emissions, with the South showing slightly higher numbers, and larger states with high treatment capacities, such as Texas, California, New York, and Florida, having the highest loadings via effluent. It is worth noting that most of the states with the highest PVA emissions, either untreated or via effluent, have coasts bordering the Pacific or Atlantic Oceans, suggesting a quicker release of PVA into aquatic or marine ecosystems. Previous research has detected wastewater-derived contaminants in the ocean [57], and similar methods could be used to trace the presence of PVA within surrounding marine or terrestrial ecosystems. As human populations and their LDP usage continue to increase, it is expected that wastewater-derived contaminants will also increase [57].

**Figure 4:**
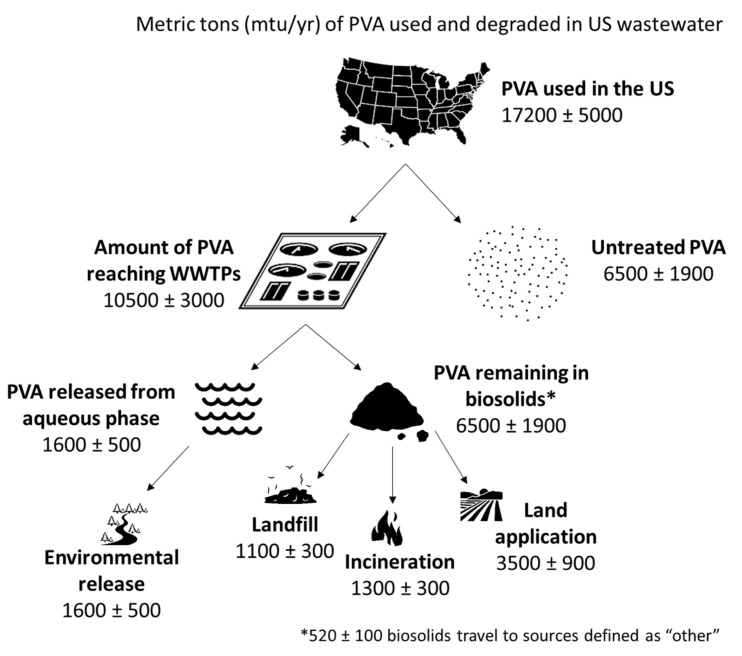
Modeled PVA usage and emissions in metric tons per year (mtu/yr) in the US.

Our data suggest that, on average, only ~10,500 ± 3000 mtu/yr (See Figure 4) of PVA enters treatment infrastructure, and only a fraction of this is biodegraded due to the specificity of conditions required to facilitate complete degradation. Based on the assumed WWTP scenario, 15.76% remains in the aqueous phase (~1600 ± 500 mtu/yr) and 61.2% (6500 ± 1900 mtu/yr) remains in the biosolids exiting the anaerobic digester. Thus, a total of 8100 ± 2400 mtu/yr of PVA is estimated to remain untreated by WWTPs annually in the United States. Of that, 6500 ± 1900 mtu/yr of PVA remains untreated due to lack of treatment capacity or inaccessibility to a functioning WWTP in certain remote communities.

Once biosolids leave a WWTP facility, 50–60% are applied to agricultural lands, 20% are sent to be incinerated, and 17% are sent to a landfill [58]. Each of these locations carries environmental risks associated with the distribution of plastics from biosolids. Initial research chronicling the negative impact of environmental PVA and future areas of study are listed in the following section. 

## 6. Implications

The pathways of sludge have been well documented, as has the ability of WWTPs to act as sources of contaminants and microplastics entering the environment. These emissions can cause deleterious impacts on surrounding ecosystems and the biota within them. PVA that passes through conventional water treatment can similarly pose a threat to the environment in several ways, once released into the environment or if land applied Our data suggest that around 3500 mtu/yr of PVA is sequestered within agricultural soils in the US. As mentioned previously, ethylene is a byproduct of PVA degradation and is also a hormone utilized by plants. It is unknown if ethylene derived from PVA could affect agricultural yields, but it warrants investigation. The ability for plastic particles to adsorb dangerous contaminants at high concentrations has been documented, but this research is currently lacking as it pertains to PVA. Initial studies revealed that PVA can alter gas exchanges, such as carbon dioxide exchange, affecting aquatic ecosystems [20]. It is also capable of leaching into the groundwater, and it has even been documented to mobilize heavy metals from sediments to water resources [59,60,61]. Hydrophilic compounds, such as biocides, insecticides, herbicides, flame retardants, corrosion inhibitors, personal care products, and pharmaceuticals are present in wastewater and stormwater [62]. Some of these are proven carcinogens [63] with great aqueous phase stability. As the sorption of organic and inorganic pollutants is not limited to hydrophobic compounds but can also occur with hydrophilic compounds, PVA could act as a vector for transport up the food chain, similarly to more conventional plastics. During such phenomena, the contaminant concentrates, increasing its level of toxicity [61]. This area requires additional research in order to further elucidate the impact of intact PVA on the natural environment.

Prior research has demonstrated that WWTPs are sources of microplastic pollution in natural and built environments. This is due to the fact that microplastics in treated sludge, termed “biosolids”, can have a variety of harmful effects on ecosystems beyond contaminant adsorption. A sizable fraction of biosolids is deposited on agricultural soils as they serve as a rich form of fertilizer, ultimately improving soil properties [64]. If biosolids are contaminated with microplastics, the particles destabilize the benefits of sludge by negatively affecting microbial activity, bulk density, and water holding capacity of the soils [57]. A portion of biosolids is also sent to landfills around the US. It is thought that this process sequesters materials in the long term, but landfill leachate has been found to carry microplastics, prompting the consideration of landfills as a source of microplastics into the environment [65]. Lastly, a portion of US biosolids is incinerated. Research has shown that incineration does not terminate plastic waste completely. On the contrary, residual ash can be considered a potential source of microplastic release into the atmosphere or environment [66]. If the plastics are completely incinerated, this process can produce airborne contaminants or pollutants [66]. As demonstrated, incomplete PVA breakdown within conventional water treatment results in a fraction of the material being sequestered within biosolids. The effects and behavior of residual PVA particles within biosolids are not well understood. More research is required to determine their impact on the environment relative to other, more conventional, plastics, whose physical presence in biosolids and ability to adsorb dangerous contaminants creates a threat to ecosystems.

In summary, this research aimed to isolate trends within the current industrial output of PVA used for laundry or dish detergent pods in US wastewater; investigate the components; assess the biodegradability, solubility, and bacterial effect on its structure; and, lastly, outline the potential risks PVA poses as an environmental pollutant. We observed that PVA has low degradation rates within WWTPs; thus, its hydrophilicity and massive production numbers make it a cause for concern as a pollutant in the natural environment. Very little research exists that aims to monitor the biodegradability of PVA in the natural environment. This presents a challenge in determining its role or impact as a pollutant. Research into truly eco-friendly substitutes for PVA is warranted and should be further explored. Improving upon this research is essential for better understanding the link between PVA usage, and public and environmental health.

## Figures and Tables

**Table 1:** Removal efficiencies of hydrophilic pharmaceuticals and their log Kow factors in filtration systems.

Compound	RE (%)	log Kow
Ibuprofen	21 [48]	2.48
Gemfibrozil	17 [48]	4.77
Diclofenac	9 [48]	1
Fenofibric acid	5 [48]	1.9
Clofibric acid	15 [48]	2.88
Carbamazepine	1.4 [49]	1.51
Doxycycline	−13 [49]	−0.62
Oxytetracycline	−33 [49]	−0.9
Sulfadiazine	7.7 [49]	−0.09
Acetaminophen	−40 [49]	0.46

**Table 2:** The treatment section, corresponding degradation percentages, SRT, HRT, and other process conditions in a conventional sewage treatment plant. Other processes that do not contribute to degradation are excluded from this table.

Sr. No.	Process	HRT (h)	SRT (days)	Other Conditions	Degradation (%)
1	Activated sludge process	18–24 [42]	12–15 [42]	F:M ratio: 0.25–0.45 [38] PVA unadapted sludge	20
2	Anaerobic digestion	NA	25 [50]	37 °C [50] PVA unadapted sludge	10
3	Disinfection	0.5 [46]	NA	chlorination 20 mgCl_2_/L	1.5

NA: Not applicable.

## Data Availability

The data presented in this study are available in the supplementary material.

## Supplementary Materials

The following are available online at https://www.mdpi.com/article/10.3390/ijerph18116027/s1, Literature review, survey questions, discussion of PVA degrading bacteria, Figure S1: Flow chart flow diagram, Table S1: Bacteria types and percent PVA degradation, Table S2: Soil PVA degradation rates, Table S3: Hydraulic retention times and solid retention times for WWTP segments, PVA Toxicity analysis.
