# Supplementary material for: Degradation of Polyvinyl Alcohol in US Wastewater Treatment Plants and Subsequent Nationwide Emission Estimate
Source: Int J Environ Res Public Health. 2021 Jun 3;18(11):6027. doi: 10.3390/ijerph18116027 (PMC8199957; doi:10.3390/ijerph18116027)
Supplement: Supplementary file 1 [file ijerph-18-06027-s001.zip › ijerph-1207913-supplementary.pdf]

Supplementary materials for

Degradation behavior of polyvinyl alcohol in US wastewater treatment plants and nationwide emission estimate

Charles Rolsky,[1,2] Varun P. Kelkar,[1,3]

Affiliations

1,2,* Biodesign Center for Environmental Health Engineering, The Biodesign Institute, Arizona State University, 1001 S. McAllister Avenue, Tempe, AZ 85287-8101, USA

2 Plastic Oceans International, Malibu, California

3 School of Sustainable Engineering and the Built Environment, Arizona State University, 660 S. College Avenue, Tempe, AZ 85281, USA

This PDF file includes:

Literature review

Survey questions

Discussion of PVA degrading bacteria

Table S1

Table S2

Table S3

Figure S1

Figure S2

PVA Toxicity analysis

References

WWTP Section	% degraded	Mechanism	Adaption
Several [1]	60-100%	NA	Adapted
Wastewater [2]	90%	Barkeri KCCM 10507 and Paenibacillus amylolyticus KCCM 10508	NA
Wastewater and Activated sludge [3]	13-21%	Pseudomonas sp	NA
Activated sludge [4]	3%	Bacillus Megaterium	NA
Activated sludge [5]	100% in 12 days	Penicillum sp.	NA
Sludge [6]	NA	Pseudomonas putida and Pseudomonas aeruginosa	Both
Activated sludge [7]	NA	Pseudomonas sp., Flavobacterium sp., Streptococcus sp. and Micrococcus sp. Pseudomonas sp., Flavobacterium sp. and Micrococcus sp.	NA
Sludge [8]	NA	Steroidobacter sp. PD	Both
Activated sludge [9]	70%	Bacillus subtilis, Pseudomonas vesicularisvar. povalolyticus	NA
Anaerobic and Aerobic bioreactor [10]	83.6-87.6%	Phyla Planctomycetes, Chlamydiae, Bacteroidetes, and Chloroflexi	NA
Activated sludge [11]	42%	Microbacterium barkeri and Paenibacillus amylolyticus	Not adapted
Wastewater [12]	85–90%	y Candida Sp. and Pseudomonas Sp	
Digestion sludge and Activated sludge [13]	7%	NA	NA
NA [14]	95%	NA	NA
Activated sludge [15]	8.9%-68,3%	Fentons reagent	NA
NA [16]	NA	Ozone	NA
Municipal sludge from anaerobic stabilization of activated sludge [17]	NA	Blend it up	Not Unadapted
Activated sludge [18]	95%	Acclimating	Adapted
Wastewater [19]	90%	"PVA-degrading bacterial strains"	NA
NA [20]	90%	Anaerobic system	NA
Anaerobically treated activated sludge [21]	60%	Digester	NA
NA [22]	91.60%	photo-oxidative degradation	NA
DI water [23]	100%	UV-365 nm/S2O8 22	NA
Wastewater [24]	91.80%	NA	NA
DI water [25]		NA	NA
Wastewater [26]	90%	NA	NA
NA [27]	38-73%	Penicillium viridicatum and Fusarium sp., Penicillium sp. and Cephalosporium sp.	NA

UV/chlorine oxidation [28]	92%	NA	NA
Municipal sewage sludge and Activated sludge [29]	7-14%	Several Pseudomonas strains	NA
NA [30]	NA	NA	NA
Culture [31]	90%	Stenotrophomonas sp. SA21	NA
Sludge, soil and water [32]	10-20%	Sphingomonas sp SA3	NA
Unknown [33]	57-74%	Fungus Phanerochaete chrysosporium	NA
Sludge from textile wastewater [34]	64-81%	B07	NA
Activated sludge [35]	NA	Bacillus niacini	NA
DI water [36]	20-80%	Pycnoporus cinnabarinus and fentons	NA

Search terms: Polyvinyl alcohol*; PVA*; Poly vinyl alcohol with results being further specified via the constraining terms (AND): Pollution*; Degradation*; Biodegradation*; Wastewater*; WWTP*; Sludge*; Sewage*; Effluent*; Influent*; Activated sludge*; US.

<u>Survey questions</u>

1 What is your gender?

2 How old are you?

3 In which state do you reside

4 What is your annual household income?

5 Please indicate the number of bedrooms and bathrooms you have in your primary residence.

6 For which of the following products are you the primary purchaser for your household? Select all that apply.

7 In the last twelve months, which of the following cleaning products have you purchased? Select all that apply.

8 How often do you purchase each of the following types of cleaning products?

9 For each of the following cleaning sprays, what size do you typically purchase?

10 For each of the following cleaning sprays, how much do you typically pay for that item? IF you do not typically purchase that item, leave it blank.

11 For Hand Soap, what size do you typically buy?

12 How much do you typically pay for Hand Soap?

13 What kind of dishwashing detergent do you currently use in your home? Select all that apply.

14 How much do you typically pay for dishwashing detergent? If you don't typically buy this item, please leave it blank

15 For liquid dish soap (hand washing), what size do you typically buy?

16 For liquid dishwasher detergent, what size do you typically buy?

17 What size powder dishwasher detergent do you typically purchase?

18 What size pack of dishwasher pods do you typically purchase?

19 What kind of laundry detergent do you currently use in your home? Select all that apply.

20 How much do you typically pay for dishwashing detergent? If you don't typically buy this item, please leave it blank

21 What size liquid laundry detergent do you typically buy?

22 What size liquid laundry detergent do you typically buy?

23 What size pack of laundry pods do you typically purchase?

24 For each of the following cleaning products, how many do you typically purchase at one time?

25 Which of the following household brands have you heard of?

26 What is the primary brand you purchase for All Purpose / Multi-Surface cleaner?

27 What is the primary brand you purchase for Glass and Mirror cleaner?

28 What is the primary brand you purchase for Bathroom cleaner?

29 What is the primary brand you purchase for Hand Soap?

30 What is the primary brand you purchase for Dish Soap (Handwashing)?

31 What is the primary brand you purchase for Liquid Dishwasher Detergent?

32 What is the primary brand you purchase for Powder Dishwasher Detergent?

33 What is the primary brand you purchase for Dishwasher Detergent Pods?

34 What is the primary brand you purchase for Liquid Laundry Detergent?

35 What is the primary brand you purchase for Powder Laundry Detergent?

36 What is the primary brand you purchase for Laundry Detergent Pods?

37 In the last twelve months, what percent of the time have you purchased cleaning products in each of the following places?

38 Do you regularly purchase these cleaning products through a subscription or auto-delivery service such as Amazon Subscribe & Save, Grove Collaborative or The Honest Company?

39 What typically determines your decision to subscribe or auto-deliver a product?

PVA Degrading Bacteria

While aqueous environments are considerably more favorable for the degradation of PVA, research suggests that it degrades poorly within terrestrial ecosystems. In soil for example, PVA has shown to only lose ~10% of its mass over a period of either 74 days or up to two years [29,37,38]. A sizable amount of variability exists due to the variety of PVA blends that are used, as well as the presence or absence of PVA-degrading microorganisms. While some studies report river water microorganisms degrading PVA, this research was conducted under anaerobic conditions, unlikely to occur in nature. Regardless, in aquatic environments, PVA will only degrade under very specific circumstances, which are unlikely to happen collectively. While in non-aquatic environments, PVA is expected to linger for considerable amounts of time, thus, its impact on the environment over long periods of time requires further study.

Table S1: List of bacteria types with corresponding percentage degraded PVA, time required and their presence in the wastewater.

**Type of bacteria**	**Degraded PVA (%)**	**Time**	**Bacterial presence in Wastewater**	**References**
Sphingopyxis sp. PVA3	90% (180 mg) degraded	6 days	Yes	[39,40]
Penicillium sp. WSH02-21	~100%	12 days	Yes	[5,31]
Sphingomonas sp. SA3 (bacterial strain SA2)	PVA1799, 95%	4 days	Yes	[32,40]
Gram-negative bacterial strain TK-2	95%	4 days	Unknown	[41]
Streptomyces venezuelae	NA	3 days	Yes	[42]
Thalassospira povalilytica sp. nov.	NA	NA	Unknown	[43]
Bacillus cereus RA23	85% of 0.1% PVA	5 days	Yes	[31]
Bacillus sp. DG22	50-75%	36 months in soil &	NA	[44]

Paenibacillus sp. DG14	24 months in soil	

Table S2: Soil medium and degradation percentages for specific PVA types and respective time durations required.

**Substrate**	**PVA type**	**% mass loss**	**Time**	**References**
Composting	PVA88 and PVA98	7%	48 days	[29]
Soil	PVA88, PVA98, PVA08/88	8–9%	74 days	[29,38]
18 soil sites in two years	PVA	Less than 10%	730 days	[37]
Sludge	PVA88 and PVA98	13%	21 days	[29]
River water microorganisms (anaerobic conditions)	PVA	50-75%	125 days	[21]

Table S3: WWTP segments with respective hydraulic retention times (HRT) and solid retention times (SRT)

WWTP segment	HRT	SRT
Gravity tank	12-24 hr	
Grit chamber	15 min	
primary clarifier	3-5 hr	
aerobic treatment	24-48 hr	5-15 days
anaerobic treatment		
Activated sludge	5-6 hr	
secondary clarifier	3-5 hr	
filters	5-10 min	
Disinfection	15-30 min	
Anaerobic Digester	72-120 days	

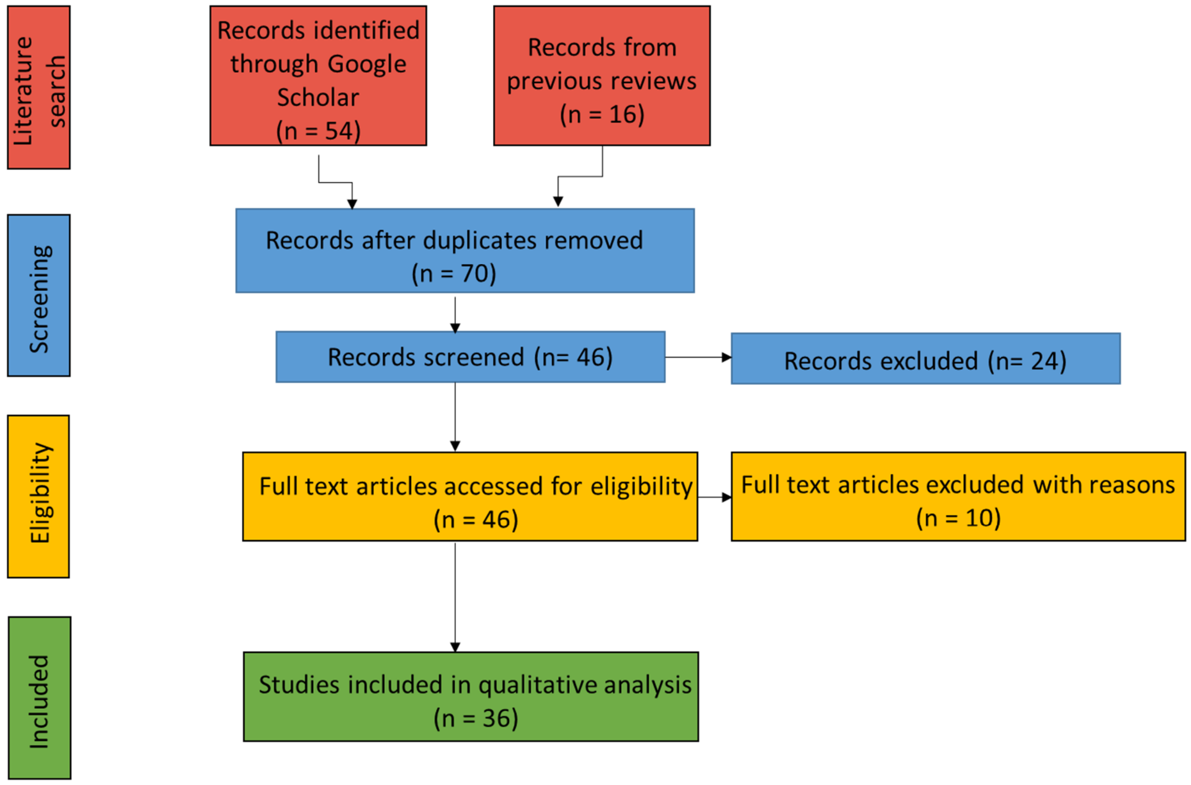

Figure S1: A flow chart for the screening of articles as they were searched and accessed in order to populate the literature review.

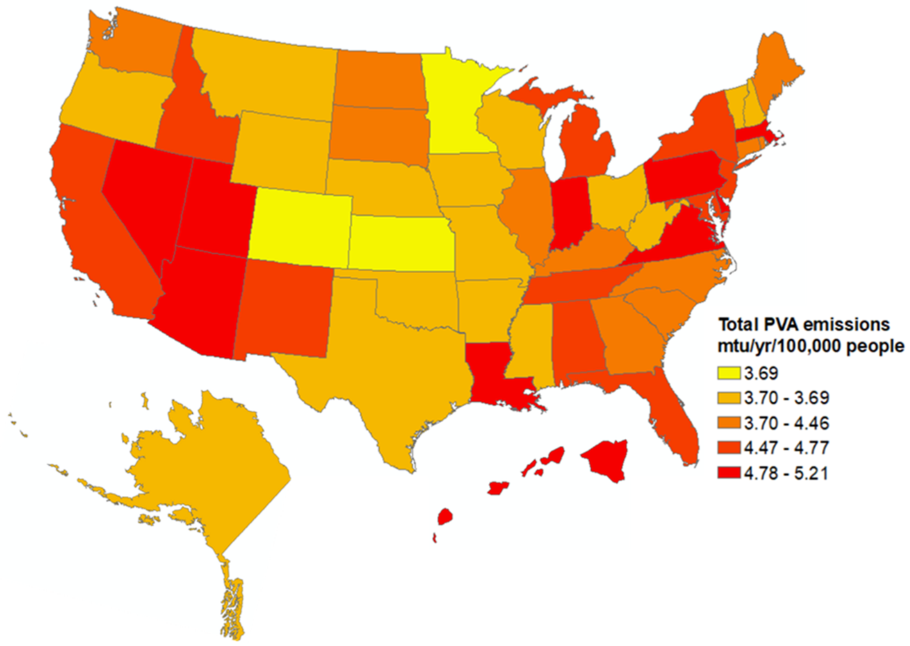

Figure S2. PVA emissions across the U.S. in mtu/yr standardized per 100,000 residents.

<u>PVA Toxicity Analysis</u>

Research has assessed the oral toxicity effects of PVA on human health due to the fact that PVA has been used widely as a coating agent for the dietary and pharmaceutical industries [45]. This research has shown medically-derived PVA to be safe for humans at normal levels. The gastrointestinal tract does not absorb PVA well and it is normally excreted through urine, in humans, once it has passed through the body [45]. Similarly, a study on rats showed that 98% of the labeled PVA orally administered was successfully recovered from the feces [45]. Other rat studies came to similar conclusions as well [46]. That being said, this area of research requires more attention as a study in 1990 by Sanders and Matthew found 0.05% of the total orally administered PVA dose accumulated in major rat tissues, such as the kidney and liver [47], suggesting that small fractions of orally administered PVA could make its way into the tissues or filtering organs and remain there. These were consistent with lower molecular weight PVA capable of traveling across membranes. A consistent, higher lethal dose of 50% (LD50) values (20,000-147,0000 mg/kg/day) for rats across multiple studies sets the limit for the safety of orally administered PVA and is assuring of relatively non-toxic nature of PVA itself [45,48–50]. To put this in perspective, an average human weighing 70 kg would have to orally consume anywhere from 1.4-103 kg/day to have adverse effect due to PVA. Since that is not possible under any imaginable scenario, PVA by itself would not be toxic to humans.

Plasticizer-Induced Toxicity

Many of the traditional plasticizers are dangerous to humans at varying concentrations [46] making exploration of plasticizers used in PVA critical. While additives of PVA have been documented previously, none of the published information on the additives themselves suggest that they are at all toxic, mutagenic, or clastogenic [45]. Some PVAs lack plasticizers all together depending upon its application. Some of the common plasticizers for PVA have been reported as Glycerin [51],Glycerol [52], water [53], ethylene glycol [54], lacti-glyceride [54], low-molecular weight polyethylene glycol (PEG) [54], MgCl2/glycol [54], urea/formamide [54], urea/ethanolamine [54], ionic liquid 1-butyl-2,3-dimethylimidazolium tetra-fluoroborate [54], citric acid, sorbitol and polyol [55]. Common carbohydrates like ribose, xylose, fructose is also commonly used [54]. All plasticizers but PEG mentioned above have been labeled as non-toxic on the SDS provided by Sigma Aldrich and would require a large dosage to cause any health issues, which seems highly unlikely under environmental scenarios. PEG is in itself a polymer and has the ability to persist in the environment unless it is being used as a carbon source by a gram-negative bacterium. Its degradation is a function of its molecular weight, with a weight less than 1000 g/mol being degraded in presence of *Pseudomonas* bacterial strain [56]. It is still unclear what molecular weight of PEG is used in PVA applications but its persistence in the environment and its ability to act as a vessel for hydrophilic contaminants and should be explored further.

References

1. Schonberger, H.; Baumann, A.; Keller, W. Study of Microbial Degradation of Polyvinyl Alcohol (PVA) in Wastewater Treatment Plants. 10.

2. Choi, K.-K.; Park, C.-H.; Kim, S.-Y.; Lyoo, W.-S.; Lee, S.-H.; Lee, J.-W. Polyvinyl Alcohol Degradation by Microbacterium barkeri KCCM 10507 and Paenibacillus amylolyticus KCCM 10508 in Dyeing Wastewater. *J. Microbiol. Biotechnol.* **2004**, *14*, 1009–1013.

3. Fukae, R.; Fujii, T.; Takeo, M.; Yamamoto, T.; Sato, T.; Maeda, Y.; Sangen, O. Biodegradation of Poly(vinyl alcohol) with High Isotacticity. *Polym. J.* **1994**, *26*, 1381–1386, doi:10.1295/polymj.26.1381.

4. Isolation and Characterization of a Strain of Bacillus megaterium That Degrades Poly(vinyl alcohol) 2021.

5. Qian, D.; Du, G.; Chen, J. Isolation and Culture Characterization of a New Polyvinyl Alcohol-Degrading Strain: Penicillium sp. WSH02-21. *World J. Microbiol. Biotechnol.* **2004**, *20*, 587, doi:10.1023/B:WIBI.0000043172.83610.08.

6. Hoffmann, J.; Řezníčková, I.; Kozáková, J.; Růžička, J.; Alexy, P.; Bakoš, D.; Precnerová, L. Assessing biodegradability of plastics based on poly(vinyl alcohol) and protein wastes. *Polym. Degrad. Stab.* **2003**, *79*, 511–519, doi:10.1016/S0141-3910(02)00367-1.

7. Chen, J.; Zhang, Y.; Du, G.-C.; Hua, Z.-Z.; Zhu, Y. Biodegradation of polyvinyl alcohol by a mixed microbial culture. *Enzyme Microb. Technol.* **2007**, *40*, 1686–1691, doi:10.1016/j.enzmictec.2006.09.010.

8. Marušincová, H.; Husárová, L.; Růžička, J.; Ingr, M.; Navrátil, V.; Buňková, L.; Koutny, M. Polyvinyl alcohol biodegradation under denitrifying conditions. *Int.*

Biodeterior. Biodegradation **2013**, *84*, 21–28, doi:10.1016/j.ibiod.2013.05.023.

9. Ishigaki, T.; Kawagoshi, Y.; Ike, M.; Fujita, M. Biodegradation of a polyvinyl alcohol-starch blend plastic film. *World J. Microbiol. Biotechnol.* **1999**, *15*, 321–327, doi:10.1023/A:1008919218289.

10. Huang, J.; Yang, S.; Zhang, S. Performance and diversity of polyvinyl alcohol-degrading bacteria under aerobic and anaerobic conditions. *Biotechnol. Lett.* **2016**, *38*, 1875–1880, doi:10.1007/s10529-016-2174-4.

11. Chung, J.; Kim, S.; Choi, K.; Kim, J.-O. Degradation of Polyvinyl Alcohol (PVA) in Textile Wastewater by Microbacterium barkeri KCCM 10507 and Paenibacillus amylolyticus KCCM 10508. *Environ. Technol.* **2015**, *37*, 1–10, doi:10.1080/09593330.2015.1054257.

12. Magdum, S.S.; Minde, G.P.; Adhyapak, U.S.; Kalyanraman, V. *An efficient biotreatment process for polyvinyl alcohol containing textile wastewater*; 2013;

13. Dvorackova, M.; Ruzicka, J.; Tu, K.D. Behavior of polyvinyl alcohol in anaerobic environment after (pre)treatment with chemical oxidation. 6.

14. Xin, D.; Xu, X.; Wang, H.; Gao, Y. Advanced oxidation of polyvinyl alcohol wastewater by O3/UV. In Proceedings of the 2011 International Symposium on Water Resource and Environmental Protection; 2011; Vol. 2, pp. 1424–1427.

15. Xiao, Y.; Xu, S.; Li, Z. Degradation of polyvinyl-alcohol wastewater by Fenton's reagent: Condition optimization and enhanced biodegradability. *J. Cent. South Univ. Technol.* **2011**, *18*, 96–100, doi:10.1007/s11771-011-0665-y.

16. Cataldo, F.; Angelini, G. Some aspects of the ozone degradation of poly(vinyl alcohol). *Polym. Degrad. Stab.* **2006**, *91*, 2793–2800, doi:10.1016/j.polymdegradstab.2006.02.018.

17. Anaerobic Biodegradation of Blends Based on Polyvinyl Alcohol | SpringerLink 2021.

18. EBSCOhost | 31744270 | Biodegradation of Polyvinyl Alcohol in Wastewater. 2021.

19. Wu, H.F.; Yue, L.Z.; Jiang, S.L.; Lu, Y.Q.; Wu, Y.X.; Wan, Z.Y. Biodegradation of polyvinyl alcohol by different dominant degrading bacterial strains in a baffled anaerobic bioreactor. *Water Sci. Technol.* **2019**, *79*, 2005–2012, doi:10.2166/wst.2019.202.

20. Russo, M.A.L.; O'Sullivan, C.; Rounsefell, B.; Halley, P.J.; Truss, R.; Clarke, W.P. The anaerobic degradability of thermoplastic starch: Polyvinyl alcohol blends: Potential biodegradable food packaging materials. *Bioresour. Technol.* **2009**, *100*, 1705–1710, doi:10.1016/j.biortech.2008.09.026.

21. Matsumura, S.; Kurita, H.; Shimokobe, H. Anaerobic biodegradability of polyvinyl alcohol. *Biotechnol. Lett.* **1993**, *15*, 749–754, doi:10.1007/BF01080150.

22. Hamad, D.; Mehrvar, M.; Dhib, R. Experimental study of polyvinyl alcohol degradation in aqueous solution by UV/H2O2 process. *Polym. Degrad. Stab.* **2014**, *103*, 75–82, doi:10.1016/j.polymdegradstab.2014.02.018.

23. Lin, C.-C.; Lee, L.-T.; Hsu, L.-J. Degradation of polyvinyl alcohol in aqueous solutions using UV-365 nm/S2O8 2− process. *Int. J. Environ. Sci. Technol.* **2014**, *11*,

831–838, doi:10.1007/s13762-013-0280-6.

24. Degradation of Polyvinyl Alcohol in Sequencing Batch Reactors: Environmental Technology: Vol 17, No 11 2021.

25. Oh, S.-Y.; Kim, H.-W.; Park, J.-M.; Park, H.-S.; Yoon, C. Oxidation of polyvinyl alcohol by persulfate activated with heat, Fe2+, and zero-valent iron. *J. Hazard. Mater.* **2009**, *168*, 346–351, doi:10.1016/j.jhazmat.2009.02.065.

26. Improvement of biodegradability of PVA-containing wastewater by ionizing radiation pretreatment | SpringerLink 2021.

27. Tudorachi, N.; Cascaval, C.N.; Rusu, M.; Pruteanu, M. Testing of polyvinyl alcohol and starch mixtures as biodegradable polymeric materials. *Polym. Test.* **2000**, *19*, 785–799, doi:10.1016/S0142-9418(99)00049-5.

28. Ye, B.; Li, Y.; Chen, Z.; Wu, Q.-Y.; Wang, W.-L.; Wang, T.; Hu, H.-Y. Degradation of polyvinyl alcohol (PVA) by UV/chlorine oxidation: Radical roles, influencing factors, and degradation pathway. *Water Res.* **2017**, *124*, 381–387, doi:10.1016/j.watres.2017.05.059.

29. Chiellini, E.; Corti, A.; Solaro, R. Biodegradation of poly(vinyl alcohol) based blown films under different environmental conditions11Part of work herewith reported was presented at the 5th Scientific Workshop on Biodegradable Polymers and Plastics, Stockholm (Se) June 1998. *Polym. Degrad. Stab.* **1999**, *64*, 305–312, doi:10.1016/S0141-3910(98)00206-7.

30. Effects of hydrogen peroxide feeding strategies on the photochemical degradation of polyvinyl alcohol: Environmental Technology: Vol 37, No 21 2021.

31. Ullah, M.; Li, H.; Sun, S.W.; Weng, C.H.; Zhang, H.; Zhu, H. Polyvinyl alcohol degradation by Bacillus cereus RA23 from oil sludge sample. *3 Biotech* **2019**, *9*, 1–8, doi:10.1007/s13205-019-1882-6.

32. Degradation of polyvinyl alcohol by Sphingomonas sp. SA3 and its symbiote | Journal of Industrial Microbiology and Biotechnology | Oxford Academic 2021.

33. Huang, M.-H.; Shih, Y.-P.; Liu, S.-M. Biodegradation of Polyvinyl Alcohol by Phanerochaete Chrysosporium After Pretreatment with Fenton's Reagent. *J. Environ. Sci. Heal. Part A* **2002**, *37*, 29–41, doi:10.1081/ESE-100108480.

34. Performance study on biodegradation simulated polyvinyl alcohol(PVA) wastewater--《Chinese Journal of Environmental Engineering》2011年01期 2021.

35. Bian, H.; Cao, M.; Wen, H.; Tan, Z.; Jia, S.; Cui, J. Biodegradation of polyvinyl alcohol using cross-linked enzyme aggregates of degrading enzymes from Bacillus niacini. *Int. J. Biol. Macromol.* **2019**, *124*, 10–16, doi:10.1016/j.ijbiomac.2018.11.204.

36. Larking, D.M.; Crawford, R.J.; Christie, G.B.Y.; Lonergan, G.T. Enhanced Degradation of Polyvinyl Alcohol byPycnoporus cinnabarinus after Pretreatment with Fenton's Reagent. *Appl. Environ. Microbiol.* **1999**, *65*, 1798–1800, doi:10.1128/AEM.65.4.1798-1800.1999.

37. Sawada, H. Field Testing of Biodegradable Plastics. In; Elsevier, 1994; Vol. 12, pp. 298–312.

38. Solaro, R.; Corti, A.; Chiellini, E. A new respirometric test simulating soil burial conditions for the evaluation of polymer biodegradation. *J. Environ. Polym. Degrad.* 1998, *6*, 203–208.

39. Yamatsu, A.; Matsumi, R.; Atomi, H.; Imanaka, T. Isolation and characterization of a novel poly(vinyl alcohol)-degrading bacterium, Sphingopyxis sp. PVA3. *Appl. Microbiol. Biotechnol.* **2006**, *72*, 804–811, doi:10.1007/s00253-006-0351-4.

40. Liu, G.; Zhu, Z.; Yang, Y.; Sun, Y.; Yu, F.; Ma, J. Sorption behavior and mechanism of hydrophilic organic chemicals to virgin and aged microplastics in freshwater and seawater. *Environ. Pollut.* **2019**, *246*, 26–33, doi:10.1016/j.envpol.2018.11.100.

41. Tokiwa, Y.; Kawabata, G.; Jarerat, A. A modified method for isolating poly(vinyl alcohol)-degrading bacteria and study of their degradation patterns. 5.

42. Zhang, Y.; Li, Y.; Shen, W.; Liu, D.; Chen, J. A new strain, Streptomyces venezuelae GY1, producing a poly(vinyl alcohol)-degrading enzyme. *World J. Microbiol. Biotechnol.* **2006**, *22*, 625–628, doi:10.1007/s11274-005-9081-5.

43. Nogi, Y.; Yoshizumi, M.; Miyazaki, M. Thalassospira povalilytica sp. nov., a polyvinyl-alcohol-degrading marine bacterium. *Int. J. Syst. Evol. Microbiol.* **2014**, *64*, 1149–1153, doi:10.1099/ijs.0.058321-0.

44. Liu, Y.; Deng, Y.; Chen, P.; Duan, M.; Lin, X.; Zhang, Y. Biodegradation analysis of polyvinyl alcohol during the compost burial course. *J. Basic Microbiol.* **2019**, *59*, 368–374, doi:10.1002/jobm.201800468.

45. DeMerlis, C.C.; Schoneker, D.R. Review of the oral toxicity of polyvinyl alcohol (PVA). *Food Chem. Toxicol.* 2003, *41*, 319–326.

46. Wei, G.L.; Li, D.Q.; Zhuo, M.N.; Liao, Y.S.; Xie, Z.Y.; Guo, T.L.; Li, J.J.; Zhang, S.Y.; Liang, Z.Q. Organophosphorus flame retardants and plasticizers: Sources, occurrence, toxicity and human exposure. *Environ. Pollut.* 2015, *196*, 29–46.

47. Sanders, J.M.; Matthews, H.B. Vaginal Absorption of Polyvinyl Alcohol in Fischer 344 Rats. *Hum. Exp. Toxicol.* **1990**, *9*, 71–77, doi:10.1177/096032719000900202.

48. Food Additives - Google Books Available online: https://www.google.com/books/edition/Food_Additives/Be4kTSsgiRYC?hl=en&gbpv=1&dq=Clydesdale,+F.+Food+Additives:+Toxicology,+Regulation,+and+Properties%3B+1996&pg=PA7&printsec=frontcover (accessed on Feb 24, 2021).

49. Zaitsev, N.A. & Sechenov, I.M. Substantiation of Hygienic Standards for Some Polymeric Compounds in Water with the Use of Gradual Standardization. *Gig. Sanit* **1986**, 75–76.

50. Burford, R.G. & Chappel, C. Range-Finding Acute Toxicity Studies of Polyvinyl Alcohol, Phthalic Acid and Cellulose Acetate Phthalate in the Mouse. **1986**.

51. Jang, J.; Lee, D.K. Plasticizer effect on the melting and crystallization behavior of polyvinyl alcohol. *Polymer (Guildf).* **2003**, *44*, 8139–8146, doi:10.1016/j.polymer.2003.10.015.

52. Rahman, W.A.W.A.; Sin, L.T.; Rahmat, A.R.; Samad, A.A. Thermal behaviour and interactions of cassava starch filled with glycerol plasticized polyvinyl alcohol blends. *Carbohydr. Polym.* **2010**, *81*, 805–810, doi:10.1016/j.carbpol.2010.03.052.

53. Park, J.-S.; Park, J.-W.; Ruckenstein, E. A dynamic mechanical and thermal analysis of unplasticized and plasticized poly(vinyl alcohol)/methylcellulose blends. *J. Appl. Polym. Sci.* **2001**, *80*, 1825–1834, doi:10.1002/app.1278.

54. Dai, H.; Wang, J.; Liu, N. Preparation and properties of poly(vinyl alcohol) films using carbohydrates as plasticizers. *J. Vinyl Addit. Technol.* **2019**, *25*, E181–E187, doi:10.1002/vnl.21679.

55. Wu, W.; Tian, H.; Xiang, A. Influence of Polyol Plasticizers on the Properties of Polyvinyl Alcohol Films Fabricated by Melt Processing. *J. Polym. Environ.* **2012**, *20*, 63–69, doi:10.1007/s10924-011-0364-7.

56. Chiellini, E.; Corti, A.; D'Antone, S.; Solaro, R. Biodegradation of poly (vinyl alcohol) based materials. *Prog. Polym. Sci.* 2003, *28*, 963–1014.
